# Controllable Synthesis of All Inorganic Lead Halide Perovskite Nanocrystals with Various Appearances in Multiligand Reaction System

**DOI:** 10.3390/nano9121751

**Published:** 2019-12-09

**Authors:** Chen Chen, Louwen Zhang, Tielin Shi, Guanglan Liao, Zirong Tang

**Affiliations:** 1 State Key Laboratory of Digital Manufacturing Equipment and Technology, Huazhong University of Science and Technology (HUST), Wuhan 430074, China; chenchen_@hust.edu.cn (C.C.); tlshi@mail.hust.edu.cn (T.S.); guanglan.liao@hust.edu.cn (G.L.); 2 Wuhan National Laboratory for Optoelectronics, Huazhong University of Science and Technology (HUST), Wuhan 430074, China; d201780047@hust.edu.cn

**Keywords:** cesium lead halide, perovskite nanocrystals, morphological control, light-emitting diodes

## Abstract

All inorganic cesium lead halide (CsPbX_3_, X = Cl, Br, I) perovskite nanocrystals (PNCs) exhibit promising applications in light-emitting devices due to their excellent photophysical properties. Herein, we developed a low-cost and convenient method for the preparation of CsPbX_3_ PNCs in a multiligand-assisted reaction system where peanut oil is applied as a ligand source. The mixed-halide PNCs with tunable optical-band gap were prepared by mixing the single-halide perovskite solutions at room temperature. The resulting PNCs had good monodispersity, with dimensions of 8–10 nm, high photoluminescence quantum yield (96.9%), narrow emission widths (15–34 nm), and tunable emission wavelength (408–694 nm), covering the entire visible spectrum. Additionally, various morphologies of PNCs, such as nanospheres, nanocubes, and nanowires, were obtained by controlling reaction temperature and time, and the amount of oleamine with multiple ligands in peanut oil potentially playing a dominant role in the nucleation/growth processes of our PNCs. Finally, the resulting CsPbBr_3_ PNCs were employed to develop a white light-emitting diode (WLED), demonstrating the potential lighting applications for our method.

## 1. Introduction

Over the past decade, organic-–inorganic hybrid perovskite has continuously attracted research attention due to its energy applications [1,2,3,4], which has, in turn, triggered a research boom on the controllable synthesis, photophysical properties, and optoelectronic devices of lead halide perovskite [5,6,7,8,9]. In 2015, Kovalenko et al. successfully synthesized all-inorganic cesium lead halide (CsPbX_3_, X = Cl, Br, I) perovskite nanocrystals (PNCs) for the first time [10]. Such PNCs possess excellent photophysical properties, such as near-unity photoluminescence quantum yield (PL QY), narrow emission-line widths, and tunable emission wavelength over the entire visible spectral region, all of which indicate their suitability for the fabrication of light-emitting devices [10,11,12,13,14,15,16]. Compared with organic–inorganic hybrid PNCs, all inorganic PNCs have better resistance to moisture and oxygen [17,18,19,20], and have performed favorably in practical applications.

So far, CsPbX_3_ PNCs have been prepared with a variety of methods [10,21,22,23,24,25,26,27], the most popular being hot injection [10], room-temperature ligand-assisted recrystallization [21], and anion exchange [22,23]. Hot injection is still the most efficient method, with high-reaction temperature and surface passivation employed to synthesize quality and uniform PNCs. In the synthesis process, oleic acid (OA) and oleamine (OAm) are typically used as surface ligands to control the growth of PNCs for a quantum-confinement effect [10]. The composition of PNCs could easily be tuned via anion exchange, which would lead to the emission light covering the whole visible spectrum. Morphological control, however, has always been an important issue in PNC research, as it determines the emission peaks of PNCs and the photophysical properties of their practical applications [28,29,30,31]. The controllable synthesis of PNCs with uniform size and morphology are still greatly needed. 

Peanut oil is a kind of edible oil that contains multiple acids. It is not only inexpensive but can also be easily obtained from most grocery shops. In this work, single-halide PNCs were successfully prepared by using more stable and cheaper peanut oil, containing multiple acids instead of the traditional oleic acid. Mixed-halide PNCs with emission wavelengths over the entire visible spectral region could be synthesized by simply mixing single-halide PNCs stock solutions at room temperature. In such a multiligand reaction system, taking cesium lead bromine PNCs as our primary example, various morphologies of PNCs, such as nanospheres, nanocubes, and nanowires, were successfully obtained under different preparation parameters. 

The most important difference from previous reports was that multiple organic ligands in peanut oil affect the nucleation/growth process of nanocrystals, resulting in different morphology results compared to the reported works with similar experiment parameters. This opens up a novel and effective route to achieving morphological control, which determines the emission peaks of PNCs and the scope of their practical applications [28,29,30,31]. Moreover, white-light-emitting diodes (WLEDs) can be further fabricated by using green CsPbBr_3_ PNCs combined with red nitride phosphors and GaN blue chips. 

## 2. Experiment Section

Chemicals: caesium carbonate (Cs_2_CO_3_, 99%), lead bromide (PbBr_2_, 99.999%), lead chloride (PbCl_2_, 99.99%), lead iodide (PbI_2_, 99.9%), trioctylphosphine (TOP, 90%), octadecene (ODE, 90%), OA (AR), OAm (80–90%), ethyl acetate (ACS, ≥99.5%) and n-hexane (anhydrous, 98%) were purchased from Aladdin Industrial Corporation, Shanghai, China. A 160 mL bottle of peanut oil was purchased from Diyidian (DYD), Zibo, China. All chemicals were directly used without further purification.

Preparation of Cs (peanut-oil precursor): Cs_2_CO_3_ (0.2 g), and peanut oil (10 mL) were loaded into a 50 mL 3-neck flask and dried for 1 h at 130 °C, and then heated under N_2_ for 2 h at 160 °C. The prepared C–peanut oil solution was stored at room temperature and preheated to 140 °C before injection. 

Synthesis of CsPbX_3_ PNCs: In a typical synthesis, 0.188 mmol PbX_2_ (0.0523 g PbCl_2_, 0.069 g PbBr_2_, or 0.867 g PbI_2_), OAm (0.5 mL), and ODE (5 mL) were loaded into a 50 mL 3-neck flask and dried under a vacuum for 1 h at 120 °C. 2 mL peanut oil was injected at 120 °C under N_2_ flow and then heated to 140 °C. Finally, the prepared Cs–peanut oil (0.4 mL) was injected into the solution, and after 1 min, the product was quickly cooled via ice-water bath. We also employed 0.5 mL TOP to promote the dissolution of PbCl_2_ powders.

Isolation and purification: 15 mL ethyl acetate was introduced into the crude PNC solution, and precipitates containing PNCs were collected by centrifuging the solution at a speed of 11,000 rpm for 6 min and redispersing in 1 mL hexane. After this, a stable colloidal NC solution was formed by centrifuging at 4000 rpm for 4 min, and the precipitates discarded.

Anion-exchange reaction: 50 μL CsPbCl_3_ PNCs or CsPbI_3_ PNCs were obtained from a 1 mL colloidal PNC solution and dispersed in 200 μL of hexane. A certain amount of diluted CsPbBr_3_ PNC solution was then introduced into a CsPbCl_3_ or CsPbI_3_ PNC solution to prepare CsPbCl_x_Br_3−x_ or CsPbBr_x_I_3−x_ PNCs. 

WLED fabrication: Green CsPbBr_3_ PNC powders, red (Sr,Ca)AlSiN_3_:Eu^2+^ phosphors, and epoxy resin were mixed via stirring. This mixture was then coated on a GaN blue LED chip, solidified in a vacuum oven at 60 °C for 30 min, and then at 90 °C for 60 min. 

Characterization: Transmission electron microscopy (TEM) and high-resolution TEM (HRTEM) images were collected by using a Tecnai G^2^ 20 U-Twin high-resolution transmission electron microscope with an acceleration voltage of 200 kV. X-ray-diffraction (XRD) patterns of NCs were measured with an Empyrean powder X-ray diffractometer (PANalytical B.V.) using Cu Kα radiation (λ = 0.15418 nm). The Fourier transform infrared (FTIR) spectrum was measured using a VERTEX 70 spectrometer produced by the Bruker Corporation. Absorption spectra were recorded using a Shimadzu UV-3600 spectrophotometer. Photoluminescence (PL) and time-resolved PL spectra were obtained using an Edinburgh FLS920 fluorescence spectrometer. The PLQY of NCs was recorded directly using a spectrofluorometer (FLS920) equipped with an integrating sphere. The electroluminescence (EL) spectra of the WLEDs were measured using a Photo Research spectroradiometer (PR655). All tests were carried out room temperature.

## 3. Results and Discussion

**Figure 1:**
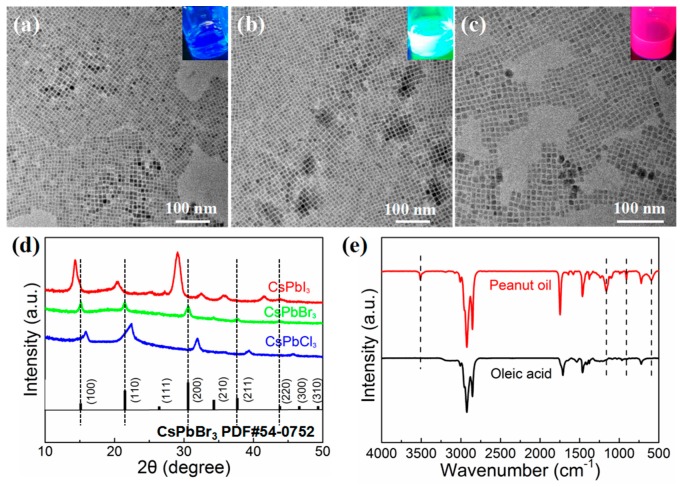
(**a**–**c**) Transmission electron microscopy (TEM) images of CsPbCl_3_, CsPbBr_3_, and CsPbI_3_ nanocrystals (NCs) prepared at 140 °C for 1 min. Insets are photographs of corresponding colloidal solution under UV irradiation. (**d**) X-ray diffraction (XRD) patterns of CsPbCl_3_, CsPbBr_3_, and CsPbI_3_ NCs. (**e**) Fourier transform infrared (FTIR) absorption spectra of CsPbBr_3_ NCs produced using peanut oil and oleic acid.

In this study, single-halide CsPbX_3_ PNCs were synthesized via a conventional hot-injection method, and slight modification was made according to the work reported by Kovalenko et al. [10]. Our detailed preparation process can be found in the Experiment Section. The resulting CsPbX_3_ PNCs showed cubic shapes with dimensions of 8–10 nm and had excellent monodispersity, as shown in Figure 1a–c. The size distribution of CsPbCl_3_, CsPbBr_3_, and CsPbI_3_ PNCs can be seen in Figure S1 in the supporting information, with average sizes of 8.36, 9.32, and 8.19 nm, respectively. CsPbX_3_ PNC solutions exhibited blue, green, and red colors, respectively, under ultraviolet (UV, λ = 365 nm) irradiation (insets shown in Figure 1a–c). HRTEM images (Figure S2) show the high crystallinity of the prepared PNCs. The selected-area electron-diffraction (SAED) pattern (Figure S3) confirmed that CsPbBr_3_ PNCs possessed the cubic structure of CsPbBr_3_ crystals with corresponding (110), (200), and (211) planes. XRD patterns were further used to determine the crystal structure of PNCs (Figure 1d). Peaks located at 15.2°, 21.6°, 30.6°, 34.4°, 37.8°, and 43.9° corresponded to (100), (110), (200), (210), (211), and (220) of the CsPbBr_3_ cubic phase (PDF#54-0752), respectively. The corresponding diffraction peaks of CsPbCl_3_ and CsPbI_3_ PNCs shifted to a certain angle at a high or low angle because the ion radius of halogen anions exhibited significant differences [10,21,22,23].

In order to confirm the existence of multiple ligands in CsPbBr_3_ PNCs synthesized using peanut oil, FTIR measurement of the thick PNC solution was performed (Figure 1e). In the FTIR spectra of the two samples with OA and peanut oil, we could see that the C-H stretching vibrations (1462, 2852, and 2926 cm^−1^) related to OA ligands are similar [32,33]. Additional absorption bands of the CsPbBr_3_ prepared with peanut oil at 595.8, 909.2, and 3511.3 cm^−1^ are ascribed to N-H_2_ characteristic absorption, which derives from other organic ligand components in peanut oil. According to previous studies, multiple ligands may exert a certain function to adjust the morphology of PNCs [34,35]. 

**Figure 2:**
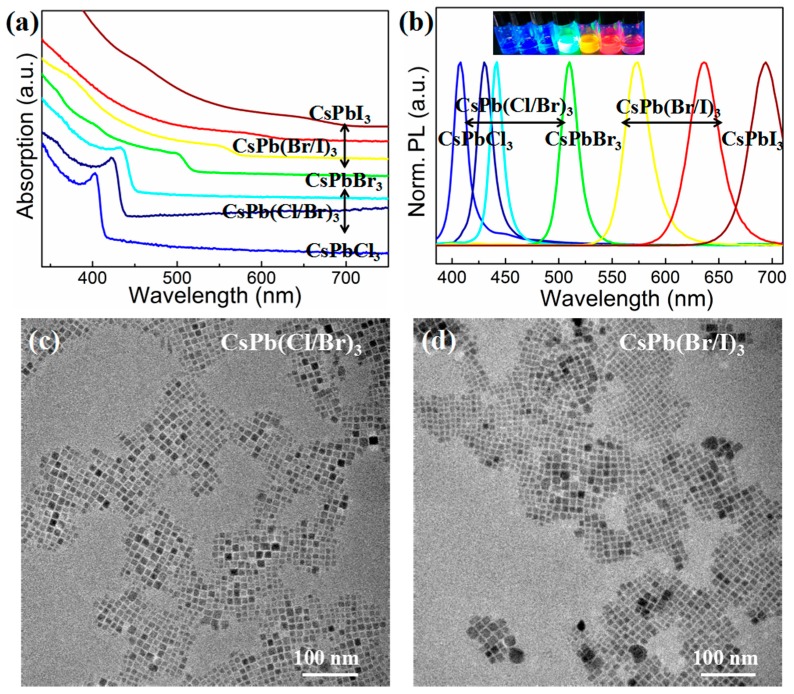
(**a**) Absorption and (**b**) photoluminescence (PL)-emission spectra of CsPbX_3_ (X = Cl, Br, I) NCs by mixing single-halide perovskite solutions. Inset of (**b**): photographs of corresponding colloidal solution under UV irradiation. TEM images of (**c**) CsPb(Cl/Br)_3_ and (**d**) CsPb(Br/I)_3_ NCs prepared via anion exchange.

The tunable emission peak of PNCs covering the entire visible spectrum can easily be calculated by controlling the ratio of halogen ions [22,23]. Here, mixed-halide PNCs were successfully prepared via anion exchange at room temperature. A series of related absorption and emission spectra are displayed in Figure 2a,b. The exciton absorption and PL emission peaks of the mixed-halide PNCs lay between the positions of CsPbBr_3_ and CsPbCl_3_ (or CsPbI_3_) PNCs when adding a certain amount of the CsPbBr_3_ solution to the CsPbCl_3_ (or CsPbI_3_) solution. The emission peak positions of CsPbCl_x_Br_3−x_ or CsPbBr_x_I_3−x_ PNCs were continuously red-shifted (408–510 nm) and blue-shifted (510–694 nm) by increasing the amount of added CsPbBr_3_ PNC solution. 

The emission peaks of CsPbCl_3_, CsPbBr_3_, and CsPbI_3_ PNCs were located at 408, 510, and 694 nm, respectively. The resulting PNCs possessed narrow emission widths (15–34 nm) and a tunable emission wavelength (408–694 nm) that shifted from the blue to the red band. Under the irradiation of a UV lamp, the prepared PNCs exhibited strong fluorescence (Figure S4 and inset of Figure 2b), and the PL QY of CsPbBr_3_ PNCs was as high as 96.9% (Table S1). The origin of the related bands was the near-band-edge emission of PNCs, which corresponded to the band gap of PNCs. One could therefore determine that PNCs do not impart severe midgap trap states [10]. 

Mixed-halide PNCs retained the cubic morphology of their parent PNCs and still achieved good monodispersity. The average sizes of CsPb(Cl/Br)_3_ and CsPb(Br/I)_3_ PNCs were 9.67 and 9.24 nm, respectively (Figure 2c,d). Figure S5 shows the excellent crystallinity of mixed-halide PNCs with peaks at 15.2°, 21.6°, and 30.6° corresponding to (100), (110), (200) of a CsPbBr_3_ cubic phase (PDF#54-0752). There was no apparent difference between the absorption and PL spectra of CsPbX_3_ PNCs prepared with OA, and those PNCs were synthesized using peanut oil, indicating that peanut oil was a viable candidate in the preparation of PNCs, but more economical and environmentally stable.

**Figure 3:**
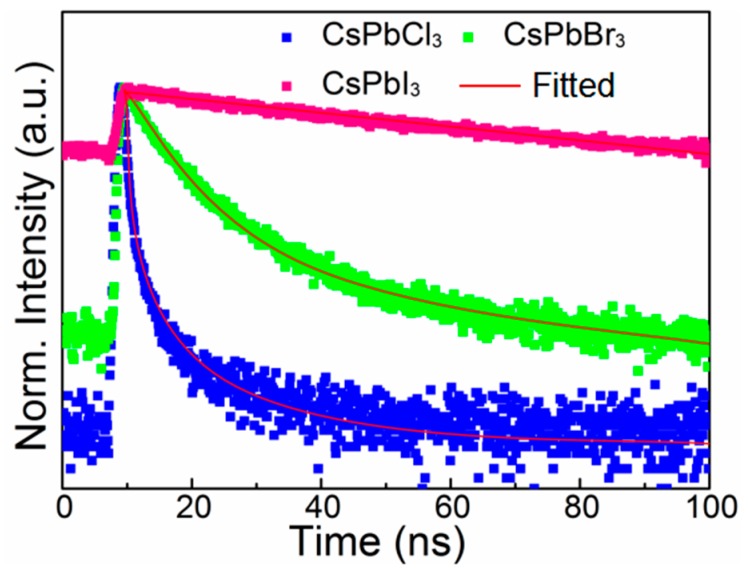
Time-resolved PL decay curves of CsPbCl_3_, CsPbBr_3_, and CsPbI_3_ NCs in hexane.

To understand the exciton recombination dynamics of PNCs prepared with peanut oil, the time-resolved PL decay measurement was calculated, as seen in Figure 3. Time-resolved PL curves were described by a bi-exponential decay function (Equation (1)). The fast-decay component (τ_1_) was relevant to trap-assisted recombination at the defect positions, and the slow-decay component (τ_2_) was associated with the radiative recombination of the NC host. The proportion (P_i_) of different components and the average lifetime (τ_ave_) were calculated as in Equations (2) and (3). 

The processing results of CsPbCl_3_, CsPbBr_3_, and CsPbI_3_ PNCs are listed in Table S2: τ_ave_ was in the range of 0.62–69.47 ns. We detected that the proportion of τ_1_ for CsPbCl_3_ PNCs was particularly large, and the proportion of τ_2_ was very small; however, the opposite was true for CsPbI_3_ PNCs. These results were consistent with previous research [10,14].

A(t) = A_1_ exp(-t/τ_1_) + A_2_ exp(-t/τ_2_), (1)

P_i_ = A_i_τ_i_/(A_1_τ_1_ + A_2_τ_2_), (2)

τ_ave_ = (A_1_τ_1_^2^ + A_2_τ_1_^2^)/(A_1_τ_1_ + A_2_τ_2_), (3)

So far, CsPbX_3_ PNCs with different shapes and sizes have been extensively studied by controlling their corresponding conditions of synthesis [28,29,30,31,34,35,36]. In this work, taking cesium lead bromine PNCs as our primary example, the influence of the experimental parameters on shape control was systematically studied, as an enormous amount of research effort has been expended on CsPbBr_3_ PNCs; therefore, they are the most representative. The change of halide composition did not affect the morphology of PNCs. Reaction temperature was an important factor to determine the morphology of the resulting products, which was closely related to PNC nucleation and growth. 

**Figure 4:**
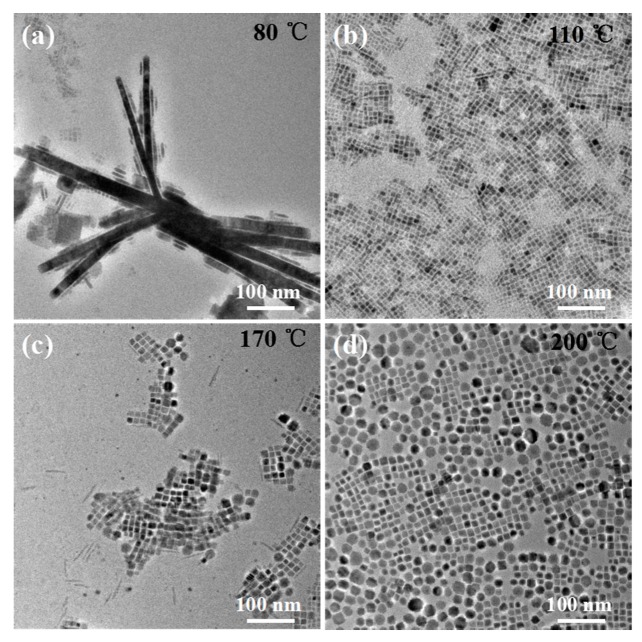
TEM images of CsPbBr_3_ NCs prepared at varying reaction temperatures: (**a**) 80 °C for 10 min, (**b**) 110 °C for 4 min, (**c**) 170 °C for 30 s, (**d**) 200 °C for 10 s.

Here, CsPbBr_3_ PNCs were prepared using peanut oil at different reaction temperatures, and their distinct morphologies were observed (Figure 4). When crystallization temperature reached 80 °C, the main products were nanorods of 25 nm width and hundreds of nanometers in length; concurrently, there were many small nanocrystals whose dimensions had not changed (Figure 4a). When the reaction temperature reached 110 °C, well-ranged cubic PNCs with 7.7 nm in size and a PL QY of 52.8% were observed, and a small number of nanowires had formed (Figure 4b). At 140 °C, unitary nanocubes with an average grain size of 9.32 nm had formed (Figure 1b). When the reaction temperature rose to 170 °C, nanocubes, nanowires, and nanospheres of mixed morphologies were produced (Figure 4c) that were quite different from the cubic morphology of PNCs prepared using OA. This was mainly because the edible peanut oil possessed multiple ligands (OA, palmitic acid, linoleic acid, etc.) leading to changes in the nucleation and growth processes of the PNCs. 

The PL QY of resulting PNCs reached as high as 96.9%. Furthermore, nanocubes and nanospheres of a mixed morphology and with an average dimension of 12.7 nm and PL QY of 91.5% had formed (Figure 4d). On the basis of experiment results as shown above, reaction temperature may have directly affected PNC morphology. The general trend of morphology evolution from a low temperature (80 °C) to high temperature (200 °C) was from nanowires, to nanocubes, and then to nanospheres. The corresponding XRD patterns can be seen in Figure S6, which matched well with the standard CsPbBr3 cubic phase indicated (PDF#54-0752). Since the above morphologies were obtained at different reaction temperatures and at different times, reaction time seemed to also play an important role in regulating the size and shape of PNCs. PNCs with different morphologies displayed different properties that would ultimately meet various application requirements [7].

**Figure 5:**
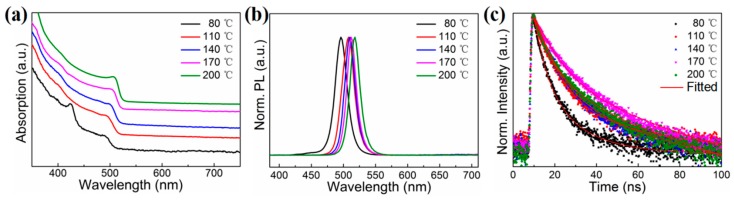
(**a**) Absorption spectra, (**b**) PL emission spectra, and (**c**) time-resolved PL decay curves of CsPbBr_3_ NCs prepared at varying reaction temperatures.

To further understand the properties of PNCs with different morphologies, the UV-visible absorption, PL, and time-resolved PL spectra were observed. The exciton absorption and PL emission peaks showed a redshift with an increase in reaction temperature from 80 °C to 200 °C (Figure 5a,b). The fluorescence emission peaks of these PNCs could be regulated in the region of 496–518 nm due to the size difference of PNCs synthesized at various temperatures [26]. The corresponding τ_ave_ first increased from 3.03 to 11.34 ns and then decreased from 11.34 to 6.52 ns with an increase in reaction temperature (Figure 5c). 

As can be seen, the proportion of τ_1_ component related to defect states in the PNCs decreased gradually as a whole (Table S3), which was consistent with the PL QY results of these PNCs. Our results indicate that an increase of reaction temperature was beneficial in the reduction of PNC defects and improved their fluorescence properties.

**Figure 6:**
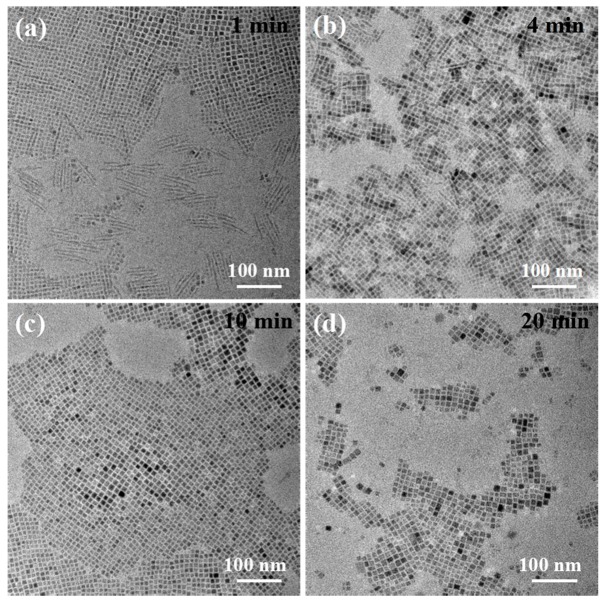
TEM images of CsPbBr_3_ NCs prepared at 110 °C for different reaction times. (**a**) 1 min, (**b**) 4 min, (**c**) 10 min, (**d**) 20 min.

Next, the effects of reaction time on the morphology and optical properties of PNCs were further studied. The mixed nanostructures of nanowires with a width of 2 nm, length of 50 nm, and nanocubes with an average size of 6.5 nm (Figure 6a) were first formed at 110 °C for 1 min. Up until 10 min in, almost all the PNCs formed were nanocubes with a size of 8.9 nm. When the reaction time was extended to 20 min, the size of the nanocubes further increased to 11.2 nm. With a longer reaction time, the morphology of PNCs changed from nanowires to nanocubes and tended to gradually stabilize. By adjusting the reaction time, the morphology and size of PNCs also had a significant effect on their optical properties (Figure S7). The photographs of the colloidal PNC solution produced at different reaction temperatures and time under UV irradiation are shown in Figure S8.

**Figure 7:**
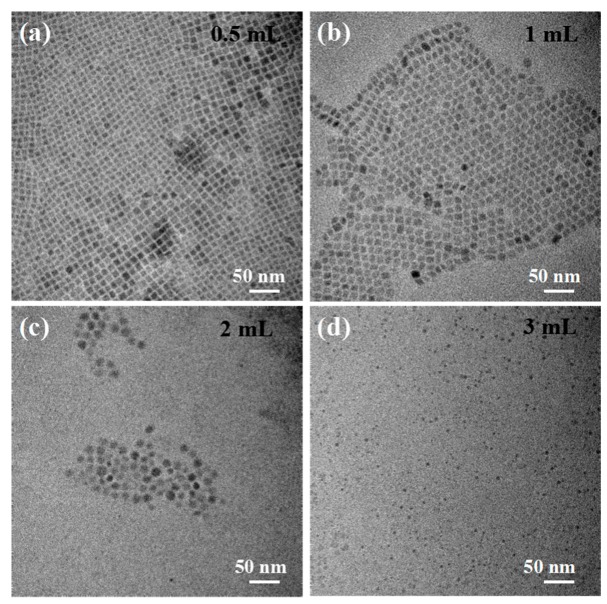
TEM images of CsPbBr_3_ PNCs prepared at 140 °C for 1 min using different amounts of oleamine (OAm): (**a**) 0.5 mL, (**b**) 1 mL, (**c**) 2 mL, (**d**) 3 mL.

Surfactant content is also one of the main factors affecting the appearance of PNCs [35]. For CsPbBr_3_ PNCs prepared using peanut oil, we studied the effect of OAm content on PNC morphology. CsPbBr_3_ PNCs were prepared at 140 °C for 1 min using different amounts of OAm, and their corresponding appearances are as seen in Figure 7. With the increase of oleamine dosage, PNC morphology changed from nanocubes to nanospheres, and their size decreased from 9.3 to 3.4 nm. This suggested that PNC morphology and size could be effectively tuned by controlling the content of OAm. 

The absorption spectra of CsPbBr_3_ NCs prepared using different amounts of OAm showed a slight blue shift (Figure S9). This was because the quantum-confinement effect of PNCs was enhanced, and the corresponding optical bandgap was increased when the size of a PNC was close to the exciton Bohr radius. Nevertheless, the small size of each PNC could have caused it to become unstable. Strong fluorescence disappeared after preservation for several days in the air when PNCs were prepared with excess OAm, as seen in Figure S10.

**Figure 8:**
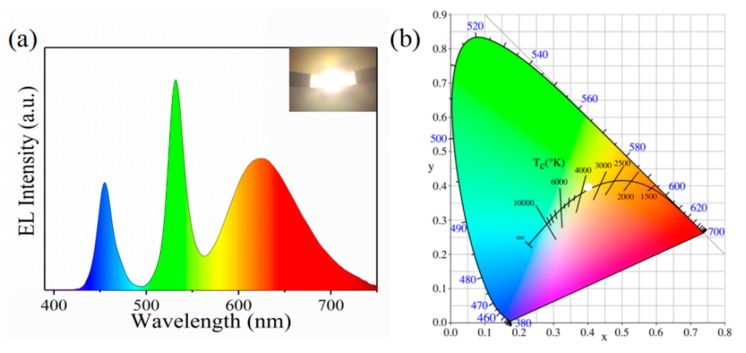
(**a**) Electroluminescence (EL) spectrum and luminescent image (inset) of as-fabricated white-light-emitting diode (WLED) under a driving current of 20 mA. (**b**) CIE color coordinates (white circle) of WLED device.

The as-synthesized green CsPbBr_3_ PNCs exhibited excellent luminescent properties, such as ultrahigh PL QY (96.9%) and narrow emission widths, which meet the application requirements of light-emitting devices. For this reason, green perovskite powder was obtained by centrifugal treatment, and images of the resulting powder are as seen in Figure S11. The green perovskite powder was then employed to fabricate WLEDs with red nitride phosphors and GaN blue chips, as shown in Figure S12. The electroluminescence spectrum of the as-fabricated WLED was as seen in Figure 8a: blue, green, and red emission peaks stemmed from LED chips, CsPbBr_3_ perovskite powder, and nitride phosphors, respectively. 

The WLED device exhibited bright white emissions (inset of Figure 8a). The WLED device had Commission Internationale de l’Eclairage (CIE) color coordinates of (0.4050, 0.3985), a color rendering index (CRI) of 81.1, and a color temperature (CCT) of 3529 K (Figure 8b). Consequently, PNCs prepared using peanut oil could have good application prospects in the field of light-emitting devices.

## 4. Conclusions

In summary, a controllable and low-cost approach was successfully proposed to synthesize CsPbX_3_ PNCs with different appearances by using peanut oil as a surface ligand. The mixed-halide PNCs with a tunable emission wavelength (408–694 nm) were obtained via anion exchange at room temperature. The experimental parameters (reaction temperature, reaction time, and the amount of OAm) had significant influence over the morphology and optical properties of PNCs; multiple ligand components in peanut oil may also have played a dominant role in the nucleation/growth processes of these PNCs. 

This work provides a Convience and novel way to prepare high-quality PNCs, and may be extended to other nanocrystalline materials, which would eventually be beneficial in the application and development of optoelectronic devices. 

## Supplementary Materials

The following are available online at https://www.mdpi.com/2079-4991/9/12/1751/s1. Figure S1. Size distribution of (a) CsPbCl_3_, (b) CsPbBr_3_, and (c) CsPbI_3_ perovskite nanocrystals (PNCs) shown in Figure 1. Figure S2. High-resolution TEM images of (a) CsPbCl_3_, (b) CsPbBr_3_, and (c) CsPbI_3_ PNCs. Figure S3. Selected area electron diffraction (SAED) patterns of CsPbBr_3_ PNCs. Figure S4. Photographs of colloidal PNC solution covering entire visible band under room light (upper) and ultraviolet lamp (bottom). Figure S5: XRD patterns of CsPb(Cl/Br)_3_ and CsPb(Br/I)_3_ PNCs prepared by anion exchange. Figure S6: XRD patterns of CsPbBr_3_ PNCs prepared at different reaction temperatures. Figure S7. (a) Absorption spectra and (b) PL emission spectra of CsPbBr_3_ PNCs prepared at 110 °C for different reaction times. Figure S8. (a) Photographs of colloidal PNC solution produced at different reaction temperatures under UV irradiation. (b) Photographs of colloidal PNC solution produced at 110 °C for different reaction times under UV irradiation. Figure S9. Absorption spectra of CsPbBr_3_ PNCs prepared at 140 °C for 1 min using different amounts of OAm. Figure S10. Photograph of colloidal PNC solution produced by excessive amount of OAm after storage in air for five days under UV irradiation. Figure S11: Images of prepared CsPbBr_3_ PNC powders (a) under room light and (b) under radiation of 365 nm UV light. Figure S12. Image of as-fabricated white light-emitting diode (WLED) device. Table S1. PL QY of CsPbX_3_ PNC solution with different reaction conditions. Table S2. Fluorescence lifetime and average lifetime for CsPbCl_3_, CsPbBr_3_, and CsPbI_3_ PNCs prepared using peanut oil. Table S3. Fluorescence lifetime and average lifetime for CsPbBr_3_ PNCs prepared at different reaction temperatures by using peanut oil.
