# Supplementary material for: Controllable Synthesis of All Inorganic Lead Halide Perovskite Nanocrystals with Various Appearances in Multiligand Reaction System
Source: Nanomaterials (Basel). 2019 Dec 9;9(12):1751. doi: 10.3390/nano9121751 (PMC6955694; doi:10.3390/nano9121751)
Supplement: Supplementary file 1 [file nanomaterials-09-01751-s001.pdf]

nanomaterials

Article

Controllable Synthesis of All Inorganic Lead Halide Perovskite Nanocrystals with Various Appearances in Multiligand Reaction System

Chen Chen [1], Louwen Zhang [2], Tielin Shi [1,2], Guanglan Liao [1,2] and Zirong Tang [1,*]

[1] State Key Laboratory of Digital Manufacturing Equipment and Technology, Huazhong University of Science and Technology (HUST), Wuhan 430074, China; chenchen_@hust.edu.cn (C.C.); tlshi@mail.hust.edu.cn (T.S.); guanglan.liao@hust.edu.cn (G.L.)

[1] Wuhan National Laboratory for Optoelectronics, Huazhong University of Science and Technology (HUST), Wuhan 430074, China; d201780047@hust.edu.cn

* Corresponding: zirong@hust.edu.cn; Tel: +8615827176117

Supplementary Materials for

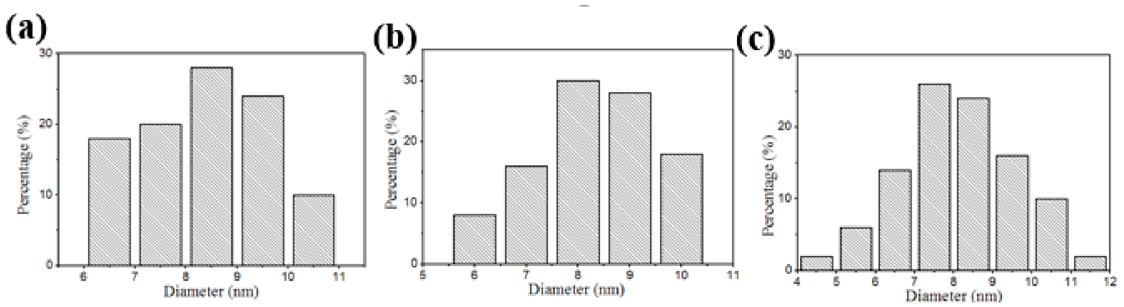

Figure 1. The size distribution of (a) $CsPbCl_3$, (b) $CsPbBr_3$, and (c) $CsPbI_3$ perovskite nanocrystals (PNCs) shown in Figure 1.

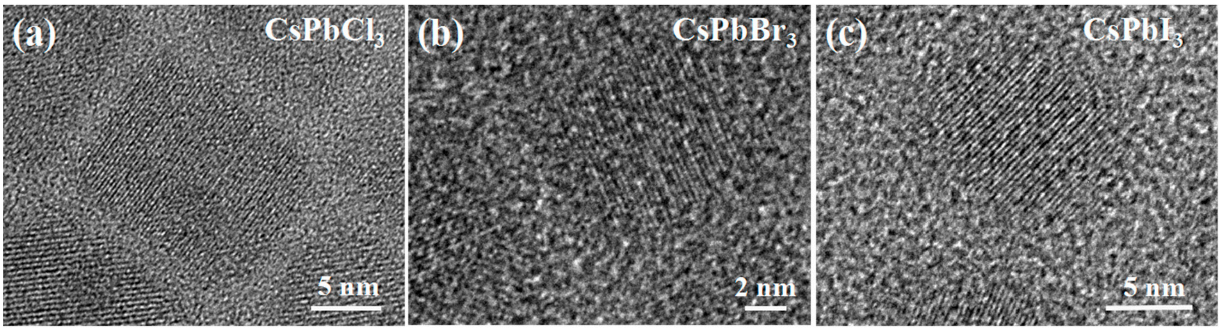

Figure 2. High-resolution TEM images of (a) $CsPbCl_3$, (b) $CsPbBr_3$, and (c) $CsPbI_3$ PNCs.

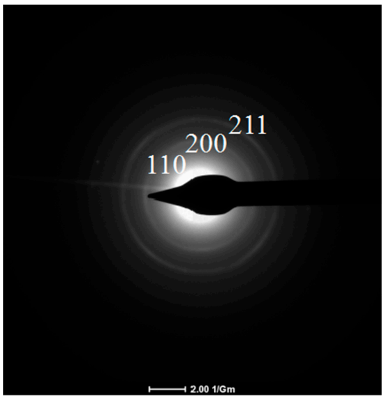

Figure 3. The selected area electron diffraction (SAED) patterns of $CsPbBr_3$ PNCs.

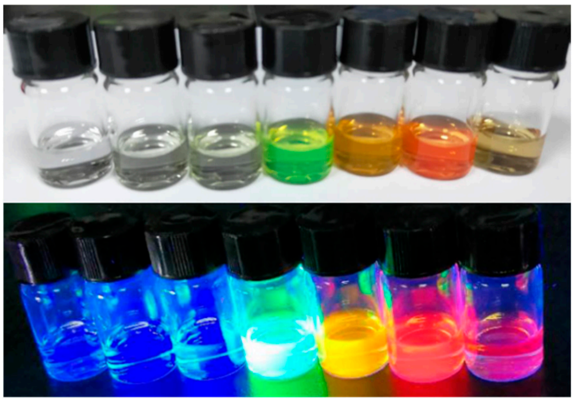

Figure 4. The photographs of the colloidal PNC solution covering the entire visible band under the room light (upper) and the ultraviolet lamp (bottom).

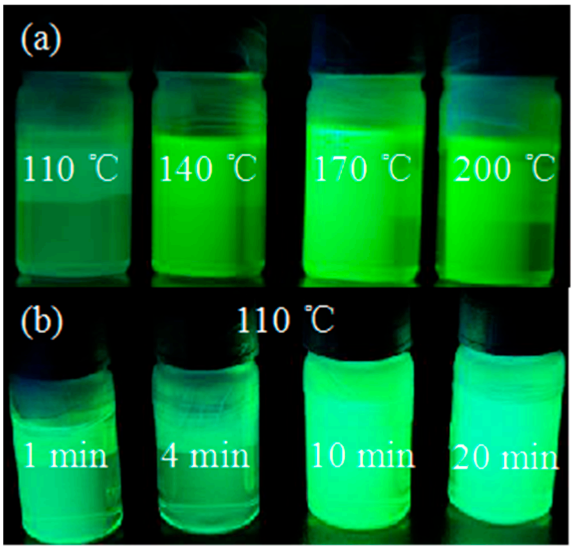

Figure S5. (a) The photographs of the colloidal PNC solution produced at different reaction temperatures under the UV irradiation. (b) The photographs of the colloidal PNC solution produced at 110 °C for different reaction times under the UV irradiation.

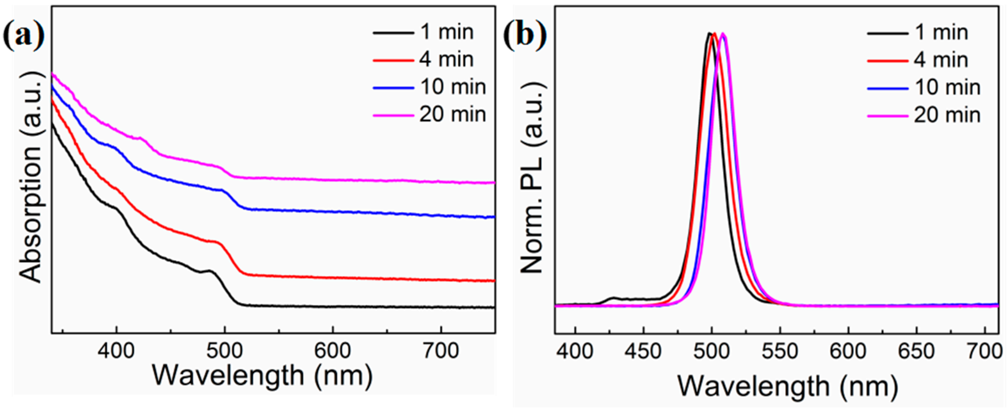

Figure 6. (a) Absorption spectra and (b) PL emission spectra of $CsPbBr_3$ PNCs prepared at 110 °C for different reaction times.

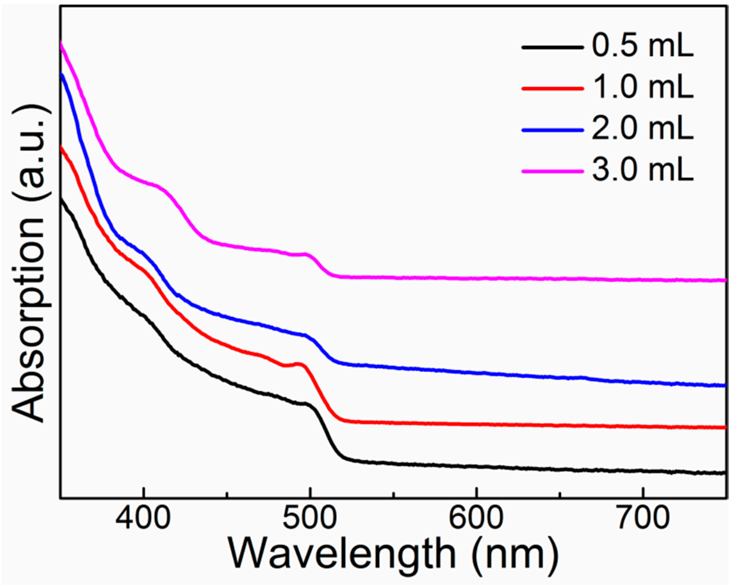

Figure 7. Absorption spectra of $CsPbBr_3$ PNCs prepared at 140 ℃ for 1 min using different amounts of oleamine (OAm).

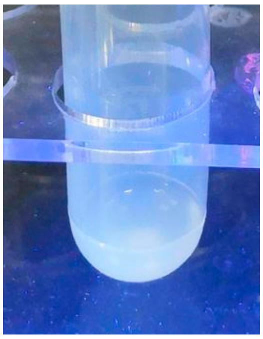

Figure 8. The photograph of the colloidal PNC solution produced by an excessive amount of OAm after storage in air for five days under the UV irradiation. This indicates that $CsPbBr_3$ PNCs are very unstable when the amount of OAm is excessive.

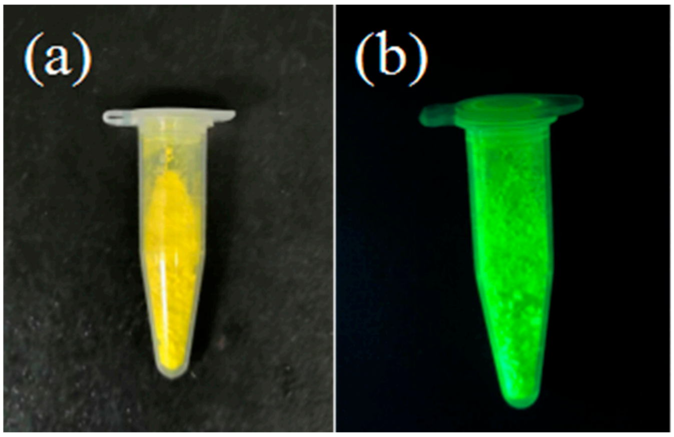

Figure 9. The images of the as-prepared $CsPbBr_3$ PNC powders. (a) Under the room light. (b) Under the radiation of 365 nm UV light.

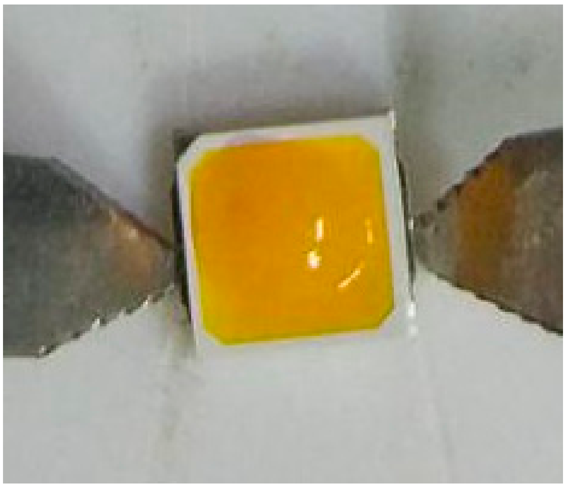

Figure 10. The image of the as-fabricated white light-emitting diode (WLED) device.

Table 1. PL QY of $CsPbX_3$ PNC solution with different reaction conditions.

Sample	Reaction temperature	Reaction time	PL QY
$CsPbBr_3$	110 ℃	4 min	52.8%
$CsPbBr_3$	140 ℃	1 min	90.3%
$CsPbBr_3$	170 ℃	30 s	96.9%
$CsPbBr_3$	200 ℃	10 s	91.5%
$CsPbBr_3$	110 ℃	10 min	78.8%
$CsPbBr_3$	110 ℃	1 min	42.3%
$CsPbI_3$	140 ℃	1 min	62.4%

Table 2. Fluorescence lifetime and average lifetime for $CsPbCl_3$, $CsPbBr_3$, and $CsPbI_3$ PNCs prepared using peanut oil.

Sample	τ_1 (ns)	τ_2 (ns)	P_1 (%)	P_2 (%)	τ_{ave} (ns)
$CsPbCl_3$	4.69	0.62	> 99.99	< 0.01	0.62
$CsPbBr_3$	4.25	14.32	70.40	29.60	7.24
$CsPbI_3$	12.95	71.29	3.10	96.90	69.47

Table 3. Fluorescence lifetime and average lifetime for $CsPbBr_3$ PNCs prepared at different reaction temperature by using peanut oil.

Reaction temperature (°C)	τ_1 (ns)	τ_2 (ns)	P_1 (%)	P_2 (%)	τ_{ave} (ns)
80	2.46	8.52	90.52	9.48	3.03
110	2.85	11.26	77.20	22.80	4.77
140	3.84	13.39	69.98	30.02	6.71
170	5.4	17.92	52.56	47.44	11.34
200	3.44	15.35	74.17	25.83	6.52

© 2019 by the authors. Submitted for possible open access publication under the terms and conditions of the Creative Commons Attribution (CC BY) license (http://creativecommons.org/licenses/by/4.0/).
